# Characterization of the Subgingival Cultivable Microbiota in Patients with Different Stages of Periodontitis in Spain and Colombia. A Cross-Sectional Study

**DOI:** 10.3390/microorganisms9091940

**Published:** 2021-09-12

**Authors:** Roquelina Pianeta, Margarita Iniesta, Diana Marcela Castillo, Gloria I. Lafaurie, Mariano Sanz, David Herrera

**Affiliations:** 1 ETEP (Etiology and Therapy of Periodontal and Peri-Implant Diseases) Research Group, Faculty of Odontology, University Complutense of Madrid (UCM), 28040 Madrid, Spain; rpianeta@ucm.es (R.P.); marsan@ucm.es (M.S.); davidher@ucm.es (D.H.); 2 School of Dentistry, Corporación Universitaria Rafael Núñez (CURN), 10003 Cartagena, Colombia; 3 Unit of Basic Oral Investigation (UIBO), School of Dentistry, Universidad El Bosque (UEB), 110121 Bogotá, Colombia; castillodiana@unbosque.edu.co (D.M.C.); lafauriegloria@unbosque.edu.co (G.I.L.)

**Keywords:** periodontitis, subgingival microbiota, microbiological culture, Spain, Colombia

## Abstract

The objective was to characterize and compare the subgingival microbiota in patients diagnosed according to the World Workshop on the Classification of Periodontal and Peri-Implant Diseases and Conditions 2018. For this cross-sectional study, Spanish and Colombian subjects (characterized as health/gingivitis, periodontitis in stages I-II or stages III-IV) were clinically assessed, and subgingival samples were taken and processed by culture. The comparisons among patients with periodontal status (and between countries) was made using Mann–Whitney, Kruskal–Wallis, ANOVA and chi-square tests. The final sample consisted of 167 subjects. *Eikenella corrodens* and *Parvimonas micra* were more frequently detected in health/gingivitis and *Porphyromonas gingivalis* in periodontitis (*p* < 0.05). Higher total counts were observed in Colombia (*p* = 0.036). In Spain, significantly higher levels of *P. gingivalis* and *Campylobacter rectus* were observed, and of *Tannerella forsythia*, *P. micra*, *Prevotella intermedia*, *Fusobacterium nucleatum*, *Actinomyces odontolyticus* and *Capnocytophaga* spp. in Colombia (*p* < 0.001). *P. micra* was more prevalent in health/gingivitis and stage I-II periodontitis in Colombia, and *P. gingivalis* in all periodontitis groups in Spain (*p* < 0.05). As conclusions, significant differences were detected in the microbiota between health/gingivitis and periodontitis, with minor differences between stages of periodontitis. Differences were also relevant between countries, with Colombia showing larger counts and variability of bacterial species.

## 1. Introduction

Periodontitis is a multifactorial chronic inflammatory disease [1,2], with a complex polymicrobial aetiology [3,4]. This disease is highly prevalent affecting large proportions of adults in different populations, depending on economic, cultural, social, and ethnic factors [5,6]. Periodontitis has recently been classified in stages and grades following the criteria of the World Workshop on the Classification of Periodontal and Peri-Implant Diseases and Conditions [1,2]. 

Bacterial communities organized in subgingival biofilms are the primary etiological factor of periodontitis [7,8]. These bacterial communities mainly result from ecological changes in their structure and the increase in total microbial biomass [3]. More than 700 different bacterial species have been identified in the subgingival microbiota [7,9,10], among which some have an effect on the general community that is much greater than the biomass they occupy, called key pathogens [3,11]. It is believed that some of these pathobionts, despite being present in low abundance in periodontitis, would produce the maturation of potentially pathogenic communities [3,4,11], which would transform a symbiotic microbiota into a dysbiotic one, breaking homeostasis and producing inflammation and tissue destruction [4,11]. 

However, studies evaluating the composition of the subgingival microbiota have resulted in a high heterogeneity, probably due to the variability in the case definitions used to select periodontitis patients [12] and sociodemographic or geographic differences [9,13], including differences in ethnicity of the populations evaluated [14] or in the age [15]. In fact, previous investigations from our research groups have reported significant differences in the prevalence and proportions of periodontal pathogens when comparing different geographical areas, using the same microbiological diagnostic techniques [13,16,17,18,19]. In these studies, a comparison between the periodontal microbiota of Dutch and Spanish patients was presented, and a lower prevalence of *Aggregatibacter actinomycetemcomitans* was observed in Spain [19]. When Spanish and Colombian patients were compared, *Porphyromonas gingivalis* was more frequent in periodontitis in Spanish patients than in Colombian patients [13]. Thus, the hypothesis arose that the present study could also be able to find differences between the studied populations, although this has to be confirmed using the criteria of the current classification.

Molecular selective techniques, including checkerboard DNA–DNA hybridization, real-time polymerase chain reaction (PCR), human oral microbe identification microarrays (HOMIM), oligonucleotide DNA-DNA hybridization, RNA-oligonucleotide quantification technique (ROQT), and techniques that allow the sequencing of the entire genome, such as pyrosequencing or next-generation sequencing (NGS) [7,12] have contributed to the characterization of periodontal microbiota, but its exclusive use could result in incomplete data on microbial diversity and, therefore, it is important to have parallel culture libraries [7,20]. Despite the fact that molecular detection techniques have greatly contributed to the knowledge of the periodontal microbiota, different studies criticize the total dependence on this type of tests, because they may underestimate a century of culture history. It has been proven that the culture is able to better detect bacterial diversity, compared to molecular methods, in addition to allowing the operator to see what has not been amplified. Perhaps, due to the analysis of the aforementioned, some researchers have turned to the use of the culture alone or in combination with other bacterial characterization techniques [7]. Due to the introduction of more competent anaerobic handling and incubation procedures, the culture is reinvented every day [17]. Thus, among many new perspectives, culture has become the basis for a high-throughput microbiological test alternative (culturomics) that have already been used in the identification of oral pathogens [21]. That is why cultivation continues to be an interesting alternative for microbiological testing [22].

The advent of the new classification of periodontitis in stages and grades [1], facilitates an adequate categorization of periodontitis patients and highlights the need of characterizing the subgingival microbiota using different microbiological methods, including anaerobic culture [21] that allows for comparisons with classical studies of prevalence and relative proportions of well-established periodontal pathogens [23]. 

Hence, the objective of the present study was to characterize and compare the subgingival cultivable microbiota, according to the criteria of the 2018 classification of periodontitis cases, in subjects from two different geographical locations (Spain and Colombia).

## 2. Materials and Methods

The present cross-sectional study was approved by the local ethical committees (references 18/127-E in Spain and 012-2018 in Colombia), and all recruited patients granted their authorization by signing the approved informed consent. All aspects of the Helsinki Declaration regarding experimentation involving human beings were considered. This manuscript follows the Strengthening the Reporting of Observational Studies in Epidemiology (STROBE) guidelines for reporting cross-sectional studies [24].

### 2.1. Subjects

The study was carried out in subjects attending the clinics of the Dental Schools at El Bosque University (UIBO) in Bogotá, Colombia, and University Complutense of Madrid (UCM), Spain. Patients were selected from April 2018 to March 2020, according to the following criteria:

#### 2.1.1. Inclusion Criteria

Adults from 30 to 60 years old were included in the study, categorized by their periodontal conditions, according to the following criteria:

- Health and gingivitis subjects, as controls. No clinical attachment loss (CAL) nor radiographical bone loss (RBL) and probing depths (PD) ≤ 3 mm, assuming no pseudo pockets [25].
- Periodontitis in stages I or II. Following the criteria for severity, interdental CAL of 1–2 mm (stage I) or 3–4 mm (stage II) and RBL affecting only the coronal third of the root (<15 % for stage I and 15–33% for stage II) [2].
- Periodontitis in stages III or IV. Following the criteria for severity, interdental CAL ≥5 mm and RBL extending to middle or apical third of the root. There should be evidence of tooth loss ≤ 4 teeth due to periodontal reasons in stage III, and ≥5 teeth in stage IV [2]. If needed, and using the criteria for complexity, furcation involvement class II or III.

#### 2.1.2. Exclusion Criteria

- Periodontal treatment in the previous year.
- Acute periodontal conditions such as periodontal abscesses or necrotizing periodontal diseases, at the time of the screening.
- Having taken antibiotics in the previous 3 months.
- Systemic diseases or conditions (diabetes, quantitative and/or qualitative polymorphonuclear neutrophil defects, other immune system disorders).
- Pregnant women.
- Chronic use of anti-inflammatories, anticonvulsants, immunosuppressants, calcium channel blockers, upon sample collection for the study, or 6 months prior to the study.

### 2.2. Study Visit

Socio-demographic variables, such as age, gender, country and smoking habits, were registered. A medical questionnaire was carried out and if inclusion and exclusion criteria were fulfilled, patients were verbally informed about the study purpose and procedures, and they were asked to participate and, upon acceptance, to sign an informed consent. Each patient received a complete periodontal and radiographic examination (panoramic and/or periapical radiographs) and microbiological samples were taken. 

### 2.3. Clinical Variables

Measurements were performed at six sites per tooth in all teeth, except third molars, with a UNC-15 periodontal probe (HuFriedy, Leinmen, Germany) by two calibrated examiners, one in each country. Clinical measurement from dental implants were not included in the analyses. All participants were clinically examined, including the following parameters, in order of registration:

- Gingival recession (REC), measured as the distance from the cementoenamel junction (CEJ) to the gingival margin and recorded to the nearest millimetre.
- PD, measured from the gingival margin to the bottom of the sulcus/pocket and recorded to the nearest millimetre.
- CAL, measured as the distance from the bottom of the pocket to the CEJ.
- Bleeding on probing (BoP), measured after probing to the base of the pocket and expressed as a percentage [26].
- Plaque index (PlI), evaluated by the presence of visible dental plaque, disclosed by erythrosine staining (Plac-Control^®^, Dentaid, Barcelona, Spain) and expressed as a percentage [27].

### 2.4. Microbiological Variables

#### 2.4.1. Microbiological Sampling

Samples were taken from four selected sites, by means of two consecutive standardized 30# sterile paper points (Maillefer, Ballaigues, Switzerland) per site. Paper points were inserted into the crevice or pocket and left in place for 10 s. Prior to sampling, supragingival plaque was removed from the sampling site followed by isolation from saliva with the use of cotton rolls and compressed air. All paper points were transferred into a screw-capped vial, containing 1.5 mL of reduced transport fluid (RTF) [28] and sent to the microbiological laboratories of each centre within 24 h. Sampling sites were selected as follows:

- In healthy/gingivitis subjects, subgingival samples were taken from the mesio-buccal sites of the first molars and, when absent, from the adjacent second molars (the next alternative was the second premolars and from there, any teeth present mesially).
- In subjects with periodontitis, subgingival samples were taken from the most accessible site with the deepest PD and BoP, per quadrant.

#### 2.4.2. Microbiological Processing

At the laboratories (UCM, UIBO), samples were homogenized by vortexing for 30 s, four serial dilutions in phosphate buffer saline (PBS) were performed, and 100 µL were placed on: (i) non-selective blood agar medium (Blood Agar Base II, Oxoid, Basingstoke, U.K.), supplemented with haemin (5 mg/L), menadione (1 mg/L) and 5%, sterile horse blood for determination of total anaerobic counts and for the identification of most target pathogens; and (ii) onto the selective medium Dentaid-1 [29] for isolation and quantification of *A. actinomycetemcomitans*. Blood agar plates were incubated for 14 days in anaerobic conditions (80%N_2_, 10% CO_2_ and 10% H_2_) at 37 °C, and selective agar plates were incubated for 3 days in air with 5% CO_2_ at 37 °C. After incubation, initial identification of target species was done using colony morphology, and suspected colonies were further identified by microscope, Gram-staining and enzyme activity. Counts of representative colonies (those with colony morphologies compatible with target pathogen morphology) were carried out. Counting was performed in plates with 30–300 colonies.

### 2.5. Statistical Analysis

#### 2.5.1. Calibration

Clinical parameters were evaluated by two calibrated examiners. An intra-examiner calibration was performed by recording duplicate measurements of PD and CAL in three patients, twice during the same visit, with 30 min intervals and intra-class reproducibility was calculated. The interclass correlation coefficient (ICC) showed an 86.3% of agreement for PD and 84.7% for CAL in Spain, and 90.2% and 89%, respectively, in Colombia.

#### 2.5.2. Sample Size Calculation

The selected outcome variable to calculate the sample size was counts of *P. gingivalis.* Based on an expected difference between healthy/gingivitis and periodontitis groups in the mean of counts (expressed as colony forming units, CFU, 2900), with a standard deviation (SD) of 3008.75 [30], an alpha error of 0.05 and a power of 90%, 30 patients per group were necessary. Besides, and in order to narrow differences between different age groups in different conditions, the overall sample followed a uniform stratified sampling, in which the same size for all the defined strata was assigned. The desired sampling distribution was 30 patients for each periodontal status group (healthy and gingivitis, periodontitis in stages I or II, periodontitis in stages III or IV), and 10 patients for each age cohort (30–40 years, 41–50 years, 51–60 years, within each periodontal status category).

#### 2.5.3. Statistical Analysis

For continuous data, Kolmogorov-Smirnov test and distribution of data were used to assess normality. Clinical and microbiological data were calculated by patient and then by periodontal status group and country. The logarithmic transformation of CFU of bacterial counts was designed to normalize the data distribution. Data were expressed as means and SD, and as median and interquartile ranges (IR) for non-parametric data. Categorical data were expressed as percentages.

Periodontal status- and country-level analyses were performed. For periodontal status-level analysis, demographic data were analysed by ANOVA test for continuous variables and chi-square test for categorical data, with probability values adjusted with the Bonferroni correction, and *p*-values were multiplied by the number of comparisons (three for periodontal status and fifteen for periodontal status by country). Clinical and microbiological variables were compared by ANOVA test, for parametric data, or Kruskal–Wallis test with Dunn–Bonferroni post hoc tests, for non-parametric data. For categorical data, chi-square test was used, with Bonferroni correction for multiplicity when was necessary. For country-level analysis, continuous demographic variables were compared with t test for parametric data, or U Mann–Whitney test for non-parametric data, and chi-square tests were used for categorical variables.

A stepwise forward logistic regression was performed to explore the relationship between the frequency of bacteria detection (yes/no) and the covariables of country, smoking, periodontal status and age. Periodontal health status (healthy/gingivitis, periodontitis stages I-II, periodontitis stages III-IV) was introduced as dummy variables. A second comparison with a stratification by age range was performed with country, smoking and periodontal status as covariables. Odds ratios (OR) together with the 95% confidence intervals (CI) were calculated. All statistical analyses were performed using SPSS 20 program package (SPSS Inc, Chicago, IL, USA) and the level of significance was set in 0.05.

## 3. Results

### 3.1. Study Population

A total of 199 patients were initially recruited, but four patients in Spain and 25 in Colombia were excluded. The reasons for exclusion in Spain were age or technical problems, and in Colombia, presenting systemic diseases, age, and having taken antibiotics in the last three months. The final sample consisted of 167 patients (Spain *n* = 90 and Colombia *n* = 77): 30 and 18, respectively, with periodontal health and gingivitis; 30 and 23, respectively, with periodontitis in stages I-II; and 30 and 36, respectively, with periodontitis in stages III-IV. In each group, three age ranges were considered: 30–40, 41–50 and 51–60 years (Table 1). Socioeconomic status was not specifically assessed. Only patients of Spanish origin (from Spain–Caucasian ethnicity) and only patients of Colombian origin (from Colombia-mixed ethnicity) were selected. Only two patients (in Spain) have received periodontal treatment in the past (subgingival instrumentation), with no supportive periodontal care for at least 3 years.

No statistically significant differences in age (*p* = 0.170), gender (*p* = 0.409) and smoking habit (*p* = 0.064), among groups according to their periodontal status, were observed (Table 2). When comparing subjects from different countries, no significant differences in age (*p* = 0.242), or gender (*p* = 0.972) were detected, but a significantly lower percentage of smokers in Colombian patients (9.1%), when compared with Spanish patients (26.7%), was evidenced (*p* = 0.004). When comparing the impact of both periodontal status and country, no significant differences were observed for age and gender, while a statistically significant higher percentage of smokers, in Spanish versus Colombian subjects, was observed in stage I-II periodontitis (30.0% versus 4.3%, respectively) (*p* = 0.031) and stage III-IV periodontitis (40.0% versus 11.1%, respectively) (*p* = 0.006), when compared to Colombian patients for the same periodontal status groups. 

### 3.2. Clinical Outcome Variables

Statistically significant differences were observed among periodontal status groups, both for mean CAL, BoP and PD, as well as for the proportion of pockets in the categories 1–3 mm and 4–5 mm (*p* < 0.001). For the proportion of deep pockets (≥6 mm), significant differences were only detected when comparing health-gingivitis with periodontitis I-II (*p* < 0.001), and periodontitis I-II with periodontitis III-IV (*p* < 0.001). PlI only presented statistically significant differences when comparing health-gingivitis with periodontitis I-II and with periodontitis III-IV (*p* < 0.001) (Table 3). The grade distribution of periodontitis did not show any difference among grades or between countries (Table S1).

When assessing periodontal status and country, statistically significant higher means in PD (*p* = 0.037) and BoP (*p* < 0.001), were observed in Colombian subjects in stages I-II periodontitis group, and statistically significant higher BoP values were also observed in stages III-IV periodontitis (*p* = 0.024). Conversely, higher PlI (*p* < 0.001) in health/gingivitis group was observed in Spain (Table 3).

Statistically significant differences were observed among periodontal status groups, both for mean CAL, BoP, and PD, as well as for the proportion of pockets in the categories 1–3 mm and 4–5 mm (*p* < 0.001). For the proportion of deep pockets (≥6 mm) significant differences were only detected when comparing health-gingivitis with periodontitis I-II (*p* < 0.001), and periodontitis I-II with periodontitis III-IV (*p* < 0.001). PlI only presented statistically significant differences when comparing health-gingivitis with periodontitis I-II and with periodontitis III-IV (*p* < 0.001) (Table 3). The clinical characteristics of the sampled sites were similar for all periodontal status and for both countries, except for PlI, which showed significantly higher levels in the health–gingivitis group in Spain than in Colombia (*p* < 0.001) (Table S2).

### 3.3. Subgingival Cultivable Microbiota-Total Anaerobic Counts

Statistically significant lower total anaerobic counts were observed for the health/gingivitis group, when compared with stages I-II periodontitis (*p* = 0.029), and stages III-IV periodontitis (*p* = 0.010). Overall, significantly higher counts were observed in Colombian patients (*p* = 0.036), together with a lower range of variability. When comparing the impact of both periodontal status and country, statistically significant higher counts were observed in Colombia for the health/gingivitis group (*p* = 0.001), when compared with Spain (Table 4).

### 3.4. Subgingival Cultivable Microbiota-Periodontal Pathogens 

Overall, the most frequently detected bacterial species were *P. gingivalis*, *Tannerella forsythia*, *Parvimonas micra*, *Capnocytophaga* spp., and *Actinomyces odontolyticus*. These pathogenic species, together with *Fusobacterium nucleatum* were also present in relatively high counts and proportions. *A. actinomycetemcomitans* was not detected in any of the samples studied. Statistically significant higher counts, proportions, and frequency of detection of *P. gingivalis* for, stages I-II periodontitis when compared with health/gingivitis (*p* ≤ 0.05), were observed. The opposite was true for *Eikenella corrodens*, with lower values and frequency of detection in stages III-IV periodontitis, when compared with health/gingivitis (*p* ≤ 0.05). In addition, *P. micra* showed statistically significant lower counts (*p* = 0.006), proportions (*p* = 0.009) and frequency of detection (*p* = 0.009), in periodontitis stages III-IV, as compared with health/gingivitis (Table 5). When comparing countries, statistically significant higher counts, proportions, and frequency of detection of *P. gingivalis* and *Campylobacter rectus* were observed in Spain (*p* < 0.001). Conversely, samples from Colombian patients showed significant higher counts, proportions, and frequency of detection of *T. forsythia*, *P. micra*, *Capnocytophaga* spp. and *A. odontolyticus* (*p* < 0.001), and counts and proportions of *Prevotella intermedia* and *F. nucleatum* (*p* ≤ 0.05) (Table 5). When assessing the impact of both periodontal status and country, for stages I-II periodontitis (Table S3), counts, and proportions of *F. nucleatum*, and proportions and frequency of detection of *P. micra*, *Capnocytophaga* spp. and *A. odontolyticus* were significantly higher in Colombian patients, compared with Spanish patients, who showed a higher frequency of detection of *P. gingivalis* (*p* < 0.001) and *P. intermedia* (*p* = 0.014), and higher counts, proportions, and detection frequencies of *E. corrodens* (*p* ≤ 0.05). For stages III-IV periodontitis (Table S4), counts, proportions and frequency of detection of *Capnocytophaga* spp. and *A. odontolyticus* were higher in Colombia, in contrast to higher frequency of detection of *P. gingivalis* (*p* ≤ 0.05) in Spain. In the health/gingivitis group (Table S5), significantly higher counts, proportions, and frequency of detection of *P. intermedia*, *T. forsythia, P. micra, Capnocytophaga* spp., and *A. odontolyticus*, in addition to higher counts of *F. nucleatum*, were observed in Colombia (*p* ≤ 0.05).

### 3.5. Impact of Periodontal Status, Country, Smoking and Age

The results of the binary logistic regression analyses are presented in Table 6 (only for species showing a reliable model). In the whole cohort, adjusted for country, smoking, periodontal status and age, *P. gingivalis* was significantly associated with Spanish subjects (OR = 10.48, 95% CI [4.59; 23.89], *p* < 0.001) in stages I-II (OR = 8.43, 95% CI [2.93; 24.19], *p* < 0.001) and stages III-IV (OR = 3.44, 95% CI [1.35; 8.78], *p* = 0.010) periodontitis. This greater probability of being detected in Spain is shown in the analysis by age strata, although the probability of *P. gingivalis* being detected in subjects from 51 to 60 years is higher in periodontitis groups than in health and gingivitis group. Conversely, *P. micra* was significantly associated with Colombian subjects (OR = 8.32, 95% CI [3.23; 21.45], *p* < 0.001) in health and gingivitis (OR = 7.68, 95% CI [2.53; 23.32], *p* < 0.001) and in stages I-II periodontitis (OR = 3.41, 95% CI [1.15; 10.05], *p* = 0.026). In addition, the probability of *P. micra* being detected increased in the 30–40-year-old stratum for Colombian subjects (OR = 10.58, 95% CI [2.33; 48.04], *p* = 0.002) with health and gingivitis (OR = 18.10, 95% CI [2.39; 137.02], *p* = 0.005) and with stages I-II periodontitis (OR = 7.91, 95% CI [1.25; 49.71], *p* = 0.027). *Capnocytophaga* spp. had also a higher probability of being detected in Colombia (OR = 19.0, 95% CI [8.72; 41.63], *p* < 0.001), and this probability is maintained in all age strata.

## 4. Discussion

The present cross-sectional study revealed differences in the subgingival microbiota between periodontitis patients (higher counts and levels of *P. gingivalis*) and healthy/gingivitis subjects (higher levels of *E. corrodens* and *P. micra*), while minor differences were observed when comparing different stages of periodontitis. Conversely, relevant differences were detected when comparing subjects from different countries, with Colombian subjects harbouring higher total counts and a larger bacterial variability than Spanish patients.

Despite the differences in terms of smoking, also reported in previous studies [13], the studied populations were comparable because the historical trends reinforce that tobacco does not seem to be a determinant in the periodontal status of the studied populations [5,31]. Despite this, the impact of smoking on the microbiological results of the present study cannot be ruled out since the impact of tobacco use in periodontitis has been clearly established [32], and may also influence the composition of the subgingival microbiota [33].

The selected populations were also comparable, since they were similar in terms of percentage of periodontal pockets and other clinical variables. All parameters showed, as expected, statistically significant differences among periodontal status groups. However, in Colombia, higher levels of BoP, specifically in periodontitis stages I-II and III-IV, and higher PD in periodontitis stages I-II were evident. In contrast, plaque levels were higher in Spain in the health/gingivitis group. As in previous studies, Colombian patients tended to present more severe clinical findings, with lower plaque levels [13]. It has been suggested that, in Colombian patients, low levels of plaque in deep pockets may be able to trigger high levels of inflammation [16], which may be conditioned by specific lifestyle conditions in developing countries [17] or by a specific genetic background in these patients [34].

Overall, the subgingival microbiota demonstrated differences between subjects with periodontal health/gingivitis (higher prevalence of *E. corrodens* and *P. micra*) and periodontitis (higher prevalence of *P. gingivalis*). While the pathogenic role of *P. gingivalis* has been clearly established as a bacterial species strongly associated with periodontitis [30], the findings for *E. corrodens* and *P. micra* are unexpected, especially for *P. micra*, which has also been considered as a periodontal pathogen with a moderate association with periodontitis [30,35,36,37].

The subgingival microbiota showed clear differences when comparing countries: Colombia, in addition to presenting higher total counts than Spain, also showed significantly higher counts, proportions, and frequency of detection of *T. forsythia, P. micra, Capnocytophaga* spp., *A. odontolyticus*, and *P. intermedia*; conversely, significant higher counts, proportions, and frequency of detection of *P. gingivalis* and *C. rectus* were found in Spain. Colombian subjects harboured higher prevalence of *P. micra* in health/gingivitis and in periodontitis stages I-II., while in Spain, higher frequencies of detection of *P. gingivalis* were observed in periodontitis stages I-II and III-IV. When also considering age stratum, *P. micra* showed a stronger association with Colombian patients in health/gingivitis and periodontitis stages I-II groups between 30 and 40 years of age. These findings suggest that the microbiota of Colombian subjects is characterized by higher bacterial counts and larger variability, while a relevant role of *P. gingivalis* in periodontitis was observed in Spanish patients, in agreement with previous studies [13,19]. A possible hypothesis may suggest that, in Colombian subjects, dysbiotic biofilms could be associated with larger amounts and greater variety in the microbiota at specific ages [38], while in Spain, the impact of key pathogens could be more relevant [39,40], regardless of age. Conducting longitudinal studies are, therefore, justified to test this hypothesis, and to better understand the possible clinical relevance and the possible impact of using microbiological diagnosis in the context of personalized medicine.

The detected differences in the periodontal microbiota between countries, according to periodontal status, could be related to the influence of specific characteristics of different geographic populations on the bacterial composition [13,16,19,41,42]. Furthermore, the 2018 classification of periodontitis does not include different types of periodontitis but rather a single multidimensional entity [23], which might be associated with different microbial profiles when comparing populations.

Thus, specific treatments and therapeutic strategies may be selected for each profile (personalized medicine).

The present study has used culture techniques to evaluate the subgingival microbiota. As explained earlier, the exclusive use of molecular techniques could result in incomplete data on microbial diversity and, therefore, it is important to have parallel culture libraries [7,20]. In addition, with the introduction of more competent anaerobic handling and incubation procedures, the culture is reinvented every day [17], and it can be still considered as a relevant tool in the characterization of periodontal pathogens [22,43,44]. Microbiological culturing is not free of limitations [7,22], but it continues to be the gold standard for bacterial identification and the starting point for current molecular analyses [7,22]. However, bacterial culture is not capable of identifying some segments of the microbiota.

Other limitations of the present study should be acknowledged: the microbiological analysis of the study samples was carried out in parallel in two independent laboratories, although they were trained and calibrated in bacterial culture [13,19,41]; the relatively small sample size; and the differences in demographic (e.g., smoking) and clinical characteristics (plaque or bleeding levels) of the population studied. In addition, no information on socioeconomic status was recorded, and patients were exclusively recruited at university clinics.

## 5. Conclusions

The present analysis, using the criteria of the 2018 classification, has observed significant differences when comparing the subgingival microbiota between periodontitis (higher levels of *P. gingivalis*) and health/gingivitis (higher levels of *E. corrodens* and *P. micra*), with minor differences between stages of periodontitis. Differences were also detected between countries, with Colombia showing larger bacterial counts and variability of bacterial species: *P. gingivalis* and *C. rectus* were the most relevant pathogens in Spain, while *T. forsythia, P. micra, P. intermedia, F. nucleatum, A. odontolyticus*, and *Capnocytophaga* spp. were the most prevalent in Colombia.

## Figures and Tables

**Table 1:** Selected subjects (*n*, number of patients) according to country, periodontal status group and age.

	Periodontal Status	Age	Spain (*n*)	Colombia (*n*)
**Selected subjects by country**			90	77
**Selected subjects by periodontal status group and country**	Health and Gingivitis		30	18
	Periodontitis I-II		30	23
	Periodontitis III-IV		30	36
**Selected subjects by periodontal status group, age and country**	Health and Gingivitis	30–40	10	7
		41–50	10	5
		51–60	10	6
	Periodontitis I-II	30–40	10	11
		41–50	10	6
		51–60	10	6
	Periodontitis III-IV	30–40	10	11
		41–50	10	16
		51–60	10	9

**Table 2:** Description of the sample population characteristics (demographic variables and smoking habit), and comparisons according to periodontal status and country.

			Age ^a^			Gender ^b^					Smoking ^b^				
Comparison	First Comparison	Second Comparison	*n*	Mean (SD)	*p* Value	*n*	Female %	*n*	Male %	*p* Value	*n*	Non Smokers %	*n*	Smokers %	*p* Value
**Periodontal status group**	Health and Gingivitis		48	45.13 (9.71)	0.170	26	54.2	22	45.8	0.409	43	89.6	5	10.4	0.064
	Periodontitis I-II		53	43.72 (8.77)		30	56.6	23	43.4		43	81.1	10	18.9	
	Periodontitis III-IV		66	45.32 (8.00)		31	47.0	35	53.0		50	75.8	16	24.2	
**Periodontal status by country**	Health and Gingivitis	Spain	30	44.97 (8.28)	0.588	16	53.3	14	46.7	1.000	27	90.0	3	10.0	0.045, Periodontitis I-II (Colombia) *versus* Periodontitis III-IV (Spain)
		Colombia	18	45.39 (11.99)		10	55.6	8	44.4		16	88.9	2	11.1	
	Periodontitis I-II	Spain	30	45.17 (8.04)		16	53.3	14	46.7		21	70.0	9	30.0	
		Colombia	23	41.83 (9.49)		14	60.9	9	39.1		22	95.7	1	4.3	
	Periodontitis III-IV	Spain	30	46.33 (8.25)		15	50.0	15	50.0		18	60.0	12	40.0	
		Colombia	36	44.47 (7.81)		16	44.4	20	55.6		32	88.9	4	11.1	

SD, standard deviation; *n*, number of patients. ^a^, One-way ANOVA test. ^b^, chi-square test with Bonferroni correction.

**Table 3:** Comparison of clinical parameters between countries (first comparison) and among periodontal status groups (second comparison).

		Health and Gingivitis ^a^			Periodontitis I-II ^a^			Periodontitis III-IV ^a^			Second Comparison (among Periodontal Status ^b^)
	First Comparison	*n*	Mean (SD)	*p* Value	*n*	Mean (SD)	*p* Value	*n*	Mean (SD)	*p* Value	*p* Value
**PD (mm)**	Spain	30	2.26 (0.31)	0.359	30	2.90 (0.39)	0.037	30	4.04 (0.76)	0.069	<0.001
	Colombia	18	2.17 (0.27)		23	3.19 (0.57)		36	3.72 (0.65)		
**PD 1–3 mm (%)**	Spain	30	98.37 (3.14)	0.148	30	77.38 (14.17)	0.073	30	43.08 (21.77)	0.083	<0.001
	Colombia	18	94.83 (12.62)		23	68.42 (21.38)		36	50.19 (9.73)		
**PD 4–5 mm (%)**	Spain	30	1.60 (3.12)	0.145	30	20.78 (12.71)	0.110	30	38.04 (14.06)	0.170	<0.001
	Colombia	18	5.16 (12.62)		23	24.07 (17.07)		36	33.41 (13.00)		
**PD ≥ 6 mm (%)**	Spain	30	0.01 (0.10)	0.445	30	1.82 (2.06)	0.174	30	18.86 (14.52)	0.424	<0.001, Health and Gingivitis *versus* Periodontitis III-IV <0.001, Periodontitis I-II *versus* Periodontitis III-IV
	Colombia	18	0		23	3.19 (4.91)		36	16.36 (10.66)		
**CAL (mm)**	Spain	30	0.33 (0.24)	0.521	30	3.07 (0.39)	0.660	30	4.78 (0.85)	0.237	<0.001
	Colombia	18	0.28 (0.26)		23	3.16 (0.96)		36	4.52 (0.93)		
**BoP (%)**	Spain	30	15.53 (15.01)	0.251	30	32.71 (17.01)	<0.001	30	63.87 (26.33)	0.024	<0.001
	Colombia	18	10.94 (9.45)		23	68.82 (38.93)		36	79.69 (28.65)		
**PlI (%)**	Spain	30	50.97 (25.07)	<0.001	30	62.69 (25.59)	0.736	30	76.00 (24.20)	0.465	<0.001, Health and Gingivitis *versus* Periodontitis I-II <0.001, Health and Gingivitis *versus* Periodontitis III-IV
	Colombia	18	22.77 (13.78)		23	65.86 (38.77)		36	70.27 (36.49)		

Probing depth (PD); clinical attachment level (CAL); bleeding on probing (BoP); plaque index (PlI). SD, standard deviation; *n*, number of patients. ^a^, Student t test. ^b^, One-way ANOVA test.

**Table 4:** Comparison of total anaerobic counts (log transformed) among periodontal status groups (first comparison) and between countries (second comparison).

Comparison	First Comparison	Second Comparison	*n*	Mean (SD)	Mean Difference	95% CI		*p* Value ^a^
						Lower Bound	Upper Bound	
**Periodontal status group**	Health and Gingivitis		48	6.29 (0.73)	−0.31 *	−0.60	−0.02	0.029
	Periodontitis I-II		53	6.60 (0.55)	−0.02 ^†^	−0.29	0.24	1.000
	Periodontitis III-IV		66	6.63 (0.53)	−0.34 ^‡^	−0.61	−0.60	0.010
**Country**		Spain	90	6.43 (0.64)	−0.20 ^§^	−0.38	−0.01	0.036
		Colombia	77	6.63 (0.56)				
**Periodontal status by country**	Health and Gingivitis	Spain	30	5.99 (0.67)	−0.80 ^§^	−1.18	−0.43	<0.001
		Colombia	18	6.79 (0.51)				
	Periodontitis I-II	Spain	30	6.57 (0.46)	−0.09 ^§^	−0.40	0.22	0.565
		Colombia	23	6.66 (0.67)				
	Periodontitis III-IV	Spain	30	6.74 (0.53)	0.20 ^§^	−0.05	0.46	0.115
		Colombia	36	6.54 (0.51)				

SD, standard deviation; *n*, number of patients; CI, confidence interval. *, health and gingivitis group *versus* periodontitis I-II group. ^†^, periodontitis I-II group *versus* periodontitis III-IV group. ^‡^, health and gingivitis group *versus* periodontitis III-IV group. ^§^, Spain *versus* Colombia. ^a^, ANOVA results in first comparison and t-test results in second comparison.

**Table 5:** Mean and standard deviation (SD) and median and interquartile range (IR) of counts (log transformed), proportions and frequencies of detection of target bacterial species according to periodontal status and country. Counts and proportions were calculated considering all samples.

	Periodontal Status (Group)	Country	Counts ^a^				Proportions ^a^				Frequency ^b^	
			*n*	Mean (SD)	Median (IR)	*p* Value	*n*	Mean (SD)	Median (IR)	*p* Value	*n* (%)	*p* Value
***Porphyromonas gingivalis***	Health and Gingivitis		48	2.34 (2.66)	0.00 (5.48)	0.014 *	48	5.88 (12.22)	0.00 (6.75)	0.007 *	22 (45.8)	0.006 *
	Periodontitis I-II		53	4.15 (2.51)	5.00 (3.77)	1.000 ^†^	53	14.57 (20.67)	3.72 (18.50)	0.512 ^†^	40 (75.5)	0.123 ^†^
	Periodontitis III-IV		66	3.38 (2.97)	5.00 (6.07)	0.062 ^‡^	66	10.28 (15.08)	3.27 (17.29)	0.195 ^‡^	38 (57.6)	0.645 ^‡^
		Spain	90	4.34 (2.33)	5.12 (1.95)	<0.001 ^§^	90	13.28 (17.52)	5.83 (18.16)	<0.001 ^§^	72 (80.0)	<0.001 ^§^
		Colombia	77	2.14 (2.88)	0.00 (5.60)		77	6.99 (14.93)	0.00 (5.06)		28 (36.4)	
***Prevotella intermedia***	Health and Gingivitis		48	3.55 (2.48)	4.15 (5.78)	0.294	48	5.59 (8.59)	0.92 (5.22)	0.497	35 (72.9)	0.978 *
	Periodontitis I-II		53	3.95 (2.11)	4.69 (2.24)		53	4.97 (8.48)	0.90 (5.66)		43 (81.1)	1.000 ^†^
	Periodontitis III-IV		66	4.17 (2.30)	5.00 (1.95)		66	7.15 (10.24)	2.33 (12.11)		52 (78.8)	1.000 ^‡^
		Spain	90	3.56 (2.14)	4.00 (2.77)	<0.001^§^	90	3.16 (6.39)	0.66 (3.33)	<0.001^§^	70 (77.8)	0.982 ^§^
		Colombia	77	4.35 (2.41)	5.43 (1.75)		77	9.35 (10.86)	5.11 (13.59)		60 (77.9)	
***Tannerella forsythia***	Health and Gingivitis		48	2.33 (2.73)	0.00 (5.58)	0.335	48	3.84 (7.10)	0.00 (5.31)	0.160	21 (43.8)	0.939 *
	Periodontitis I-II		53	1.72 (2.45)	0.00 (4.65)		53	0.89 (1.87)	0.00 (0.79)		18 (34.0)	1.000 ^†^
	Periodontitis III-IV		66	1.83 (2.47)	0.00 (4.81)		66	0.87 (1.77)	0.00 (1.02)		24 (36.4)	1.000 ^‡^
		Spain	90	1.40 (2.18)	0.00 (4.30)	<0.001 ^§^	90	0.57 (1.63)	0.00 (0.65)	<0.001 ^§^	27 (30.0)	0.026 ^§^
		Colombia	77	2.57 (2.78)	0.00 (5.60)		77	3.09 (5.79)	0.00 (3.70)		36 (46.8)	
***Parvimonas micra***	Health and Gingivitis		48	1.77 (2.57)	0.00 (5.03)	0.503 *	48	2.34 (6.27)	0.00 (2.41)	0.633 *	16 (33.3)	1.000 *
	Periodontitis I-II		53	1.19 (2.23)	0.00 (0.00)	0.275 ^†^	53	1.57 (4.12)	0.00 (0.00)	0.271 ^†^	12 (22.6)	0.225 ^†^
	Periodontitis III-IV		66	0.52 (1.55)	0.00 (0.00)	0.006 ^‡^	66	0.57 (2.54)	0.00 (0.00)	0.009 ^‡^	7 (10.6)	0.009 ^‡^
		Spain	90	0.42 (1.36)	0.00 (0.00)	<0.001 ^§^	90	0.28 (1.59)	0.00 (0.00)	<0.001 ^§^	8 (8.9)	<0.001 ^§^
		Colombia	77	1.88 (2.61)	0.00 (5.14)		77	2.70 (6.04)	0.00 (3.26)		27 (35.1)	
***Fusobacterium nucleatum***	Health and Gingivitis		48	4.48 (1.24)	4.69 (1.13)	0.111	48	3.63 (3.37)	2.58 (4.19)	0.951	46 (95.8)	0.822 *
	Periodontitis I-II		53	4.52 (1.74)	5.04 (1.08)		53	4.66 (5.53)	2.45 (5.15)		47 (88.7)	1.000 ^†^
	Periodontitis III-IV		66	4.54 (1.91)	5.00 (1.14)		66	8.24 (13.55)	2.65 (9.20)		58 (87.9)	0.564 ^‡^
		Spain	90	4.16 (1.58)	4.60 (1.04)	<0.001 ^§^	90	2.58 (2.90)	1.95 (2.97)	<0.001 ^§^	81 (90.0)	0.842 ^§^
		Colombia	77	4.94 (1.71)	5.39 (1.19)		77	9.52 (12.57)	4.17 (9.06)		70 (90.9)	
***Campylobacter rectus***	Health and Gingivitis		48	0.23 (0.91)	0.00 (0.00)	0.876	48	0.18 (0.88)	0.00 (0.00)	0.849	3 (6.3)	1.000 *
	Periodontitis I-II		53	0.20 (1.03)	0.00 (0.00)		53	0.53 (3.42)	0.00 (0.00)		2 (3.8)	1.000 ^†^
	Periodontitis III-IV		66	0.20 (0.94)	0.00 (0.00)		66	0.07 (0.37)	0.00 (0.00)		3 (4.5)	1.000 ^‡^
		Spain	90	0.39 (1.28)	0.00 (0.00)	<0.001 ^§^	90	0.46 (2.70)	0.00 (0.00)	<0.001 ^§^	8 (8.9)	0.008 ^§^
		Colombia	77	0.00 (0.00)	0.00 (0.00)		77	0.00 (0.00)	0.00 (0.00)		0 (0.0)	
***Eikenella corrodens***	Health and Gingivitis		48	1.03 (1.89)	0.00 (2.25)	0.048 *	48	0.35 (0.91)	0.00 (0.09)	0.039 *	12 (25.0)	0.051 *
	Periodontitis I-II		53	0.37 (1.18)	0.00 (0.00)	1.000 ^†^	53	0.07 (0.37)	0.00 (0.00)	1.000 ^†^	5 (9.4)	1.000 ^†^
	Periodontitis III-IV		66	0.47 (1.53)	0.00 (0.00)	0.074 ^‡^	66	0.45 (1.85)	0.00 (0.00)	0.066 ^‡^	6 (9.1)	0.033 ^‡^
		Spain	90	0.66 (1.53)	0.00 (0.00)	0.343 ^§^	90	0.19 (0.75)	0.00 (0.00)	0.355 ^§^	15 (16.7)	0.241 ^§^
		Colombia	77	0.53 (1.60)	0.00 (0.00)		77	0.43 (1.70)	0.00 (0.00)		8 (10.4)	
***Capnocytophaga* spp.**	Health and Gingivitis		48	2.36 (2.74)	0.00 (5.43)	0.727	48	3.30 (6.31)	0.00 (4.24)	0.889	21 (43.8)	1.000 *
	Periodontitis I-II		53	2.55 (2.77)	0.00 (5.30)		53	4.94 (11.23)	0.00 (4.47)		25 (47.2)	1.000 ^†^
	Periodontitis III-IV		66	2.31 (2.61)	0.00 (5.12)		66	4.14 (11.22)	0.00 (3.33)		30 (45.5)	1.000 ^‡^
		Spain	90	0.74 (1.68)	0.00 (0.00)	<0.001 ^§^	90	0.15 (0.63)	0.00 (0.00)	<0.001 ^§^	15 (16.7)	<0.001 ^§^
		Colombia	77	4.34 (2.31)	5.30 (1.39)		77	8.83 (13.34)	4.17 (9.34)		61 (79.2)	
***Actinomyces odontolyticus***	Health and Gingivitis		48	2.24 (2.94)	0.00 (5.71)	0.374	48	6.95 (10.96)	0.00 (12.56)	0.278	18 (37.5)	1.000 *
	Periodontitis I-II		53	2.14 (2.81)	0.00 (5.37)		53	5.82 (9.97)	0.00 (10.62)		20 (37.7)	0.399 ^†^
	Periodontitis III-IV		66	2.14 (2.81)	4.15 (5.63)		66	10.48 (17.67)	0.65 (16.12)		34 (51.5)	0.414 ^‡^
		Spain	90	0.00 (0.00)	0.00 (0.00)	<0.001 ^§^	90	0.00 (0.00)	0.00 (0.00)	<0.001 ^§^	0 (0.00)	<0.001 ^§^
		Colombia	77	5.35 (1.59)	5.69 (1.24)		77	17.33 (15.95)	13.87 (20.47)		72 (93.5)	

*n*, number of patients; *n* (%), number and percentage of positive samples. *, health and gingivitis group versus periodontitis I-II group; ^†^, periodontitis I-II group versus periodontitis III-IV group; ^‡^, health and gingivitis group versus periodontitis III-IV group; ^§^, Spain versus Colombia. ^a^, Kruskal–Wallis test and Mann–Whitney test. ^b^, chi-square with Bonferroni correction.

**Table 6:** Forward stepwise logistic regression analyses evaluating the detection of selected bacterial species considering country, smoking, periodontal status, and age as covariables in a first comparison with the whole cohort, and in a second comparison stratified by age ranges.

	Adjusted Model			Coefficient	*p* Value	OR	95% CI	
	Whole Cohort	Stratification by Age Ranges						
***Porphyromonas gingivalis***	Spain			2.35	<0.001	10.48	4.59	23.89
	Periodontitis I-II			2.13	<0.001	8.43	2.93	24.19
	Periodontitis III-IV			1.23	0.010	3.44	1.35	8.78
		30–40 years	Spain	2.02	0.001	7.60	2.34	24.67
		41–50 years	Spain	1.91	0.002	6.80	2.07	22.29
		51–60 years	Spain	2.52	0.003	12.4	2.36	65.45
			Periodontitis I-II	2.69	0.010	14.7	1.90	114.05
			Periodontitis III-IV	2.08	0.027	8.01	1.26	50.83
***Parvimonas micra***	Colombia			2.12	<0.001	8.32	3.23	21.45
	Health and gingivitis			2.03	<0.001	7.68	2.53	23.32
	Periodontitis I-II			1.22	0.026	3.41	1.15	10.05
		30–40 years	Colombia	2.35	0.002	10.58	2.33	48.04
			Health and Gingivitis	2.89	0.005	18.1	2.39	137.02
			Periodontitis I-II	2.06	0.027	7.91	1.25	49.71
***Capnocytophaga*** **spp.**	Colombia			2.94	<0.001	19.0	8.72	41.63
		30–40 years	Colombia	3.44	<0.001	31.2	7.50	130.17
		41–50 years	Colombia	2.07	0.001	8.00	2.40	26.56
		51–60 years	Colombia	3.66	<0.001	39.0	7.72	195.71

OR, odds ratio; CI, confidence interval.

## Data Availability

Data available on request due to restrictions. Data presented in this study are available on request from the corresponding author. The data are not publicly available due to privacy and ethical issues.

## Supplementary Materials

The following are available online at https://www.mdpi.com/article/10.3390/microorganisms9091940/s1, Table S1: Distribution of periodontitis grades by country. Table S2: Clinical characteristics of the selected sites for subgingival sampling. Table S3: Mean and standard deviation (SD) and median and interquartile range (IR) of counts (log transformed), proportions and frequencies of detection of target bacterial species according to country, in periodontitis in stages I-II patients. Table S4: Mean and standard deviation (SD) and median and interquartile range (IR) of counts (log transformed), proportions and frequencies of detection of target bacterial species according to country, in periodontitis in stages III-IV patients. Table S5: Mean and standard deviation (SD) and median and interquartile range (IR) of counts (log transformed), proportions and frequencies of detection of target bacterial species, according to country, in health and gingivitis subjects.
