# Supplementary material for: Characterization of the Subgingival Cultivable Microbiota in Patients with Different Stages of Periodontitis in Spain and Colombia. A Cross-Sectional Study
Source: Microorganisms. 2021 Sep 12;9(9):1940. doi: 10.3390/microorganisms9091940 (PMC8469102; doi:10.3390/microorganisms9091940)
Supplement: Supplementary file 1 [file microorganisms-09-01940-s001.zip › microorganisms-1338112-supplementary/microorganisms-1338112-supplementary.pdf]

microorganisms

Article

Characterization of the Subgingival Cultivable Microbiota in Patients with Different Stages of Periodontitis in Spain and Colombia. A Cross-Sectional Study

Roquelina Pianeta [1,2], Margarita Iniesta [1,*], Diana Marcela Castillo [3], Gloria I. Lafaurie [3], Mariano Sanz [1] and David Herrera [1]

[1] ETEP (Etiology and Therapy of Periodontal and Peri-implant Diseases) Research Group, Faculty of Odontology, University Complutense of Madrid (UCM), 28040 Madrid, Spain; rpianeta@ucm.es (R.P.); marsan@ucm.es (M.S.); davidher@ucm.es (D.H.)
[2] School of Dentistry, Corporación Universitaria Rafael Núñez (CURN), Cartagena 10003, Colombia
[3] Unit of Basic Oral Investigation (UIBO), School of Dentistry, Universidad El Bosque (UEB), Bogotá 110121, Colombia; castillodiana@unbosque.edu.co (D.M.C.); lafauriegloria@unbosque.edu.co (G.I.L.)
* Correspondence: margaini@ucm.es

Citation: Pianeta, R.; Iniesta, M.; Castillo, D.M.; Lafaurie, G.I.; Sanz, M.; Herrera, D. Characterization of the Subgingival Microbiota in Patients with Different Stages of Periodontitis in Spain and Colombia. A Cross-Sectional Study. *Microorganisms* **2021**, *9*, 1940. https://doi.org/10.3390/microorganisms9091940

Academic Editor: Martin Von Bergen

Received: 28 July 2021
Accepted: 11 September 2021
Published: 12 September 2021

Publisher's Note: MDPI stays neutral with regard to jurisdictional claims in published maps and institutional affiliations.

Copyright: © 2021 by the authors. Licensee MDPI, Basel, Switzerland. This article is an open access article distributed under the terms and conditions of the Creative Commons Attribution (CC BY) license (http://creativecommons.org/licenses/by/4.0/).

Table S1. Distribution of periodontitis grades by country.

		Spain		Colombia			Second comparison (between countries[a])
	**First comparison**	**n**	**%**	**n**	**%**	***p* value**	***p* value**
	A	8	13.3	7	11.9		
**Grade**	B	24	40.0	26	44.1	1.000	1.000
	C	28	46.7	26	44.1		

n, number of patients; %, percentage. [a], Chi-square test with Bonferroni correction.

Periodontitis grades:

Indirect estimation was made using bone loss as a function of age at the most affected tooth or case presentation (radiographic bone loss (RBL) expressed as percentage of root length divided by the age of the subject, RBL/age):

- Periodontitis in grade A. Percentage of RBL / age <0.25, non-smoker.
- Periodontitis in grade B. Percentage of RBL / age 0.25 to 1.0, smoker <10 cigarettes/day.
- Periodontitis in grade C. Percentage of RBL / age >1.0, smoker ⩾10 cigarettes/day.

Table S2. Clinical characteristics of the selected sites for subgingival sampling.

		Health and Gingivitis			Periodontitis I-II			Periodontitis III-IV		
		**n**	**Mean (SD)**	***p* value**[a]	**n**	**Mean (SD)**	***p* value**[a]	**n**	**Mean (SD)**	***p* value**[a]
**PD (mm)**	Spain	30	2.79 (0.39)	0.271	30	4.43 (0.69)	0.203	30	6.10 (1.29)	0.280
	Colombia	18	2.66 (0.33)		23	4.71 (0.91)		36	5.79 (1.01)	
**CAL (mm)**	Spain	30	0	1.000	30	4.09 (0.31)	0.411	30	6.47 (1.31)	0.824
	Colombia	18	0		23	3.92 (1.05)		36	6.39 (1.52)	
**BoP (%)**	Spain	30	36.66 (40.86)	0.060	30	87.50 (21.52)	0.111	30	95.00 (13.77)	0.158
	Colombia	18	15.27 (29.87)		23	95.65 (12.27)		36	98.61 (5.80)	
**PlI (%)**	Spain	30	55.00 (32.43)	<0.001	30	80.00 (28.16)	0.126	30	93.33 (15.99)	0.070
	Colombia	18	9.72 (22.91)		23	91.30 (23.36)		36	98.61 (5.80)	

Probing depth (PD); clinical attachment level (CAL); bleeding on probing (BoP); plaque index (PlI). SD, standard deviation; n, number of patients.

[a], Student t test.

Table S3. Mean and standard deviation (SD) and median and interquartile range (IR) of counts (log transformed), proportions and frequencies of detection of target bacterial species according to country, in periodontitis in stages I-II patients. Counts and proportions were calculated considering all samples.

		Periodontitis I-II									
		Counts[a]				Proportions[a]				Frequency[b]	
	Country	n	Mean (SD)	Median (IR)	*p* value	n	Mean (SD)	Median (IR)	*p* value	n (%)	*p* value
Porphyromonas gingivalis	Spain	30	5.07 (1.20)	5.05 (0.92)	0.161	30	13.89 (18.78)	7.27 (14.33)	0.068	29 (96.7)	<0.001
	Colombia	23	2.95 (3.22)	0.00 (6.26)		23	15.45 (23.32)	0.00 (36.00)		11 (47.8)	
Prevotella intermedia	Spain	30	4.15 (1.41)	4.32 (1.57)	0.238	30	2.32 (4.16)	0.82 (2.34)	0.189	28 (93.3)	0.014
	Colombia	23	3.70 (2.79)	5.30 (5.78)		23	8.43 (11.19)	5.06 (12.50)		15 (65.2)	
Tannerella forsythia	Spain	30	1.39 (2.18)	0.00 (4.30)	0.184	30	0.39 (0.79)	0.00 (0.67)	0.251	9 (30.0)	0.487
	Colombia	23	2.15 (2.76)	0.00 (5.26)		23	1.54 (2.58)	0.00 (2.86)		9 (39.1)	
Parvimonas micra	Spain	30	0.32 (1.22)	0.00 (0.00)	0.001	30	0.49 (2.64)	0.00 (0.00)	0.002	2 (6.7)	0.002
	Colombia	23	2.32 (2.73)	0.00 (5.30)		23	2.96 (5.23)	0.00 (4.69)		10 (43.5)	
Fusobacterium nucleatum	Spain	30	4.21 (1.75)	4.69 (0.98)	0.008	30	2.52 (2.86)	1.99 (3.02)	0.004	26 (86.7)	0.687
	Colombia	23	4.94 (1.68)	5.34 (1.12)		23	7.46 (6.85)	6.69 (9.70)		21 (91.3)	
Campylobacter rectus	Spain	30	0.35 (1.36)	0.00 (0.00)	0.211	30	0.94 (4.54)	0.00 (0.00)	0.211	2 (6.7)	0.499
	Colombia	23	0	0		23	0	0		0 (0.0)	
Eikenella corrodens	Spain	30	0.66 (1.51)	0.00 (0.00)	0.042	30	0.14 (0.48)	0.00 (0.00)	0.042	5 (16.7)	0.061
	Colombia	23	0	0		23	0	0		0 (0.0)	
***Capnocytophaga* spp.**	Spain	30	0.78 (1.80)	0.00 (0.00)	<0.001	30	0.13 (0.36)	0.00 (0.00)	<0.001	5 (16.7)	<0.001
	Colombia	23	4.85 (2.02)	5.47 (1.30)		23	11.21 (15.02)	6.12 (11.97)		20 (87.0)	
Actinomyces odontolyticus	Spain	30	0	0	<0.001	30	0	0	<0.001	0 (0.0)	<0.001
	Colombia	23	4.94 (2.07)	5.63 (1.34)		23	13.42 (11.32)	13.86 (16.80)		20 (87.0)	

n, number of patients; n (%), number and percentage of positive samples. [a], Mann-Whitney test. [b], Chi-square test.

Table S4. Mean and standard deviation (SD) and median and interquartile range (IR) of counts (log transformed), proportions and frequencies of detection of target bacterial species according to country, in periodontitis in stages III-IV patients. Counts and proportions were calculated considering all samples.

		Periodontitis III-IV									
	Country	Counts[a]				Proportions[a]				Frequency[b]	
		n	Mean (SD)	Median (IR)	*p* value	n	Mean (SD)	Median (IR)	*p* value	n (%)	*p* value
Porphyromonas gingivalis	Spain	30	5.34 (1.94)	5.97 (1.34)	<0.001	30	18.27 (17.98)	13.72 (25.91)	<0.001	27 (90.0)	<0.001
	Colombia	36	1.74 (2.69)	0.00 (5.00)		36	3.63 (7.38)	0.00 (3.70)		11 (30.6)	
Prevotella intermedia	Spain	30	4.27 (2.09)	5.00 (1.99)	0.736	30	5.09 (8.45)	1.65 (3.84)	0.263	25 (83.3)	0.410
	Colombia	36	4.09 (2.48)	5.20 (4.83)		36	8.87 (11.36)	3.53 (13.90)		27 (75.0)	
Tannerella forsythia	Spain	30	2.11 (2.48)	0.00 (4.68)	0.515	30	0.80 (1.73)	0.00 (0.89)	0.638	13 (43.3)	0.283
	Colombia	36	1.60 (2.47)	0.00 (4.94)		36	0.94 (1.81)	0.00 (1.21)		11 (30.6)	
Parvimonas micra	Spain	30	0.48 (1.48)	0.00 (0.00)	0.885	30	0.09 (0.37)	0.00 (0.00)	0.772	3 (10.0)	1.000
	Colombia	36	0.56 (1.63)	0.00 (0.00)		36	0.97 (3.39)	0.00 (0.00)		4 (11.1)	
Fusobacterium nucleatum	Spain	30	4.25 (1.69)	4.77 (1.00)	0.012	30	1.68 (1.63)	0.99 (2.30)	<0.001	27 (90.0)	0.719
	Colombia	36	4.79 (2.07)	5.42 (1.29)		36	13.72 (16.47)	8.76 (21.59)		31 (86.1)	
Campylobacter rectus	Spain	30	0.45 (1.37)	0.00 (0.00)	0.054	30	0.17 (0.54)	0.00 (0.00)	0.054	3 (10.0)	0.089
	Colombia	36	0	0		36	0	0		0 (0.0)	
Eikenella corrodens	Spain	30	0.68 (1.79)	0.00 (0.00)	0.296	30	0.35 (1.18)	0.00 (0.00)	0.326	4 (13.3)	0.399
	Colombia	36	0.30 (1.27)	0.00 (0.00)		36	0.53 (2.28)	0.00 (0.00)		2 (5.6)	
***Capnocytophaga* spp.**	Spain	30	1.02 (1.90)	0.00 (0.90)	<0.001	30	0.10 (0.20)	0.00 (0.05)	<0.001	7 (23.3)	0.001
	Colombia	36	3.39 (2.65)	5.00 (5.42)		36	7.51 (14.42)	1.71 (8.54)		23 (63.9)	
Actinomyces odontolyticus	Spain	30	0	0	<0.001	30	0	0	<0.001	0 (0.0)	<0.001
	Colombia	36	5.31 (1.52)	5.60 (1.52)		36	19.23 (20.17)	11.40 (26.99)		34 (94.4)	

n, number of patients; n (%), number and percentage of positive samples. [a], Mann-Whitney test. [b], Chi-square test.

Table S5. Mean and standard deviation (SD) and median and interquartile range (IR) of counts (log transformed), proportions and frequencies of detection of target bacterial species, according to country, in health and gingivitis subjects. Counts and proportions were calculated considering all samples.

		Health and Gingivitis									
		**Counts[a]**				**Proportions[a]**				**Frequency[b]**	
	**Country**	**n**	**Mean (SD)**	**Median (IR)**	***p* value**	**n**	**Mean (SD)**	**Median (IR)**	***p* value**	**n (%)**	***p* value**
Porphyromonas gingivalis	Spain	30	2.62 (2.61)	3.29 (5.42)	0.444	30	7.67 (14.42)	0.43 (10.28)	0.178	16 (53.3)	0.178
	Colombia	18	1.88 (2.76)	0.00 (5.63)		18	2.90 (6.56)	0.00 (2.28)		6 (33.3)	
Prevotella intermedia	Spain	30	2.26 (2.24)	2.23 (4.00)	<0.001	30	2.05 (5.55)	0.03 (0.72)	<0.001	17 (56.7)	0.001
	Colombia	18	5.68 (0.81)	5.77 (1.18)		18	11.47 (9.64)	7.61 (12.89		18 (100. 0)	
Tarnerella forsythia	Spain	30	0.72 (1.65)	0.00 (0.00)	<0.001	30	0.52 (2.12)	0.00 (0.00)	<0.001	5 (16.7)	<0.001
	Colombia	18	5.03 (1.92)	5.77 (0.88)		18	9.38 (8.94)	6.75 (12.74)		16 (88.9)	
Parvimonas micra	Spain	30	0.45 (1.41)	0.00 (0.00)	<0.001	30	0.24 (0.79)	0.00 (0.00)	<0.001	3 (10.0)	<0.001
	Colombia	18	3.97 (2.59)	5.26 (5.79)		18	5.84 (9.33)	3.35 (5.89)		13 (72.2)	
Fusobacterium nucleatum	Spain	30	4.03 (1.29)	4.25 (1.03)	<0.001	30	3.54 (3.63)	2.39 (3.40)	0.443	28 (93.3)	0.521
	Colombia	18	5.23 (0.65)	5.25 (0.91)		18	3.78 (2.99)	2.73 (5.05)		18 (100)	
Campylobacter rectus	Spain	30	0.37 (1.14)	0.00 (0.00)	0.171	30	0.29 (1.11)	0.00 (0.00)	0.171	3 (10.0)	0.282
	Colombia	18	0	0		18	0	0		0 (0.0)	
Eikenella corrodens	Spain	30	0.64 (1.31)	0.00 (0.00)	0.134	30	0.08 (0.23)	0.00 (0.00)	0.138	6 (20.0)	0.325
	Colombia	18	1.69 (2.49)	0.00 (4.68)		18	0.80 (1.37)	0.00 (1.51)		6 (33.3)	
***Capnocytophaga* spp.**	Spain	30	0.42 (1.28)	0.00 (0.00)	<0.001	30	0.23 (1.02)	0.00 (0.00)	<0.001	3 (10.0)	<0.001
	Colombia	18	5.59 (0.49)	5.59 (0.78)		18	8.42 (8.00)	5.06 (7.39)		18 (100.0)	
Actinomyces odontolyticus	Spain	30	0	0	<0.001	30	0	0	<0.001	0 (0.00)	<0.001
	Colombia	18	5.97 (0.60)	5.99 (0.95)		18	18.54 (10.23)	21.21 (15.54)		18 (100.0)	

n, number of patients; n (%), number and percentage of positive samples. [a], Mann-Whitney test. [b], Chi-square test.
